# In Vitro Evaluation of PCL and P(3HB) as Coating Materials for Selective Laser Melted Porous Titanium Implants

**DOI:** 10.3390/ma10121344

**Published:** 2017-11-23

**Authors:** Michael Grau, Julia Matena, Michael Teske, Svea Petersen, Pooyan Aliuos, Laura Roland, Niels Grabow, Hugo Murua Escobar, Nils-Claudius Gellrich, Heinz Haferkamp, Ingo Nolte

**Affiliations:** 1 Small Animal Clinic, University of Veterinary Medicine Hannover, Foundation, D-30559 Hannover, Germany; michael.grau@tiho-hannover.de (M.G.); julia.matena@gmx.de (J.M.); laura.roland@yahoo.de (L.R.); hugo.murua.escobar@med.uni-rostock.de (H.M.E.); 2 Division of Medicine Clinic III, Hematology, Oncology and Palliative Medicine, University of Rostock, D-18057 Rostock, Germany; 3 Institute for Biomedical Engineering, Rostock University Medical Center, D-18119 Rostock, Germany; michael.teske@uni-rostock.de (M.T.); niels.grabow@uni-rostock.de (N.G.); 4 Faculty of Engineering and Computer Science, University of Applied Sciences, D-49076 Osnabrueck, Germany; s.petersen@hs-osnabrueck.de; 5 Department of Otorhinolaryngology, Head and Neck Surgery, Hannover Medical School, D-30625 Hannover, Germany; paliuos@yahoo.com; 6 Clinic for Cranio-Maxillo-Facial Surgery, Hannover Medical School, D-30625 Hannover, Germany; gellrich.nils-claudius@mh-hannover.de; 7 Institut fuer Werkstoffkunde, Leibniz Universitaet Hannover, D-30823 Garbsen, Germany; haferkamp@iw.uni-hannover.de

**Keywords:** titanium scaffold, polycaprolactone, poly(3-hydroxybutyrate), osteoblast

## Abstract

Titanium is widely used as a bone implant material due to its biocompatibility and high resilience. Since its Young’s modulus differs from bone tissue, the resulting “stress shielding” could lead to scaffold loosening. However, by using a scaffold-shaped geometry, the Young’s modulus can be adjusted. Also, a porous geometry enables vascularisation and bone ingrowth inside the implant itself. Additionally, growth factors can improve these effects. In order to create a deposit and release system for these factors, the titanium scaffolds could be coated with degradable polymers. Therefore, in the present study, synthetic poly-ε-caprolactone (PCL) and the biopolymer poly(3-hydroxybutyrate) (P(3HB)) were tested for coating efficiency, cell adhesion, and biocompatibility to find a suitable coating material. The underlying scaffold was created from titanium by Selective Laser Melting (SLM) and coated with PCL or P(3HB) via dip coating. To test the biocompatibility, Live Cell Imaging (LCI) as well as vitality and proliferation assays were performed. In addition, cell adhesion forces were detected via Single Cell Force Spectroscopy, while the coating efficiency was observed using environmental scanning electron microscopy (ESEM) and energy-dispersive X-ray (EDX) analyses. Regarding the coating efficiency, PCL showed higher values in comparison to P(3HB). Vitality assays revealed decent vitality values for both polymers, while values for PCL were significantly lower than those for blank titanium. No significant differences could be observed between PCL and P(3HB) in proliferation and cell adhesion studies. Although LCI observations revealed decreasing values in cell number and populated area over time on both polymer-coated scaffolds, these outcomes could be explained by the possibility of coating diluent residues accumulating in the culture medium. Overall, both polymers fulfill the requirements regarding biocompatibility. Nonetheless, since only PCL coating ensured the maintenance of the porous implant structure, it is preferable to be used as a coating material for creating a deposit and release system for growth factors.

## 1. Introduction

While titanium is widely used as a biocompatible bone implant material, the long-term side effects of stress shielding and the resulting implant loosening due to the difference in Young’s moduli of titanium and bone are still given [1,2,3]. These challenges can be overcome by creating porous scaffold-shaped implants whose Young’s modulus is not only influenced by the underlying material, but also by their porosity. Thus, an annealing of the scaffold’s Young’s modulus to the one of bone material is possible by varying its strut width and thereby its pore size [4].

By using Selective Laser Melting (SLM^®^), it is possible to create highly complex implants [5]. Since this process enables the production of filigree yet stable shapes, SLM has become well established for manufacturing titanium implants for bone defect treatment [6,7,8]. For optimal osteoblast activity and therefore proper osteogenesis, pore sizes of 20–1500 µm have been discussed recently [1]. A strut and pore size of 230 µm showed promising mechanical results in force loading femur defects while acting osteoconductively [9]. Also, pore sizes of 160–270 µm are reported to support fast and broad vascularisation, which is needed for nutrition of newly built tissue [9].

In order to enhance bone defect healing, even further titanium-based scaffolds could be coated with a polymeric layer that may be able to act as a deposit and release system for specific growth factors as recently described [10]. A positive effect on angiogenesis of the growth factors VEGF and HMGB-1 being incorporated into polymer-coated titanium implants was demonstrated in a previous study of ours [11]. As the coating material should not just be tested for biocompatibility like any other new biomaterial [12] but should also fulfill properties like high coating efficiency and strong cell adhesion, this study deals with the overall suitability of certain polymers as coating materials for SLM-produced titanium scaffolds. Synthetic poly-ε-caprolactone (PCL) was chosen due to the fact that it is an already established material for bone implants [13,14]. The biologically produced poly-3-hydroxybutyrate (P(3HB)) was selected to act as a possible alternate coating material since it has also demonstrated good properties as bone implant material [15,16]. 

This study mainly dealt with two aspects of the polymer coating. One focus was on coating efficiency in order to find a material that meets the requirements to act as a deposit and release system for growth factors. However, no experiments on drug release were performed within this publication. To ensure a usage of the implants within prospective in vivo studies, a further important aspect of this study was the testing of the polymers’ biocompatibility. SLM-produced porous titanium scaffolds (pore size: 250 µm) coated with PCL or P(3HB) without incorporated biologically active substances were compared with uncoated implants of the same geometry. To gain an impression of the coating efficiency, environmental scanning electron microscopy (ESEM) and energy-dispersive X-ray (EDX) measurements were performed. Murine green fluorescent protein (GFP)-linked osteoblasts were then chosen to vitalise the hybrid construct since they are commonly used for evaluating the cytocompatibility of biomaterials in vitro due to their representation of bony tissue in the skull [17]. Since bone implants are meant to be integrated in the osseous tissue, tests with direct cell–implant contact were selected in this study. Firstly, to compare the cell behaviour on the differently coated implants, Live Cell Imaging (LCI) was performed as it provides the opportunity to track viable cells on the nontransparent implant surface by following the cell’s morphology and migratory behaviour as recently reported [18,19]. Secondly, to test viability and proliferation of osteoblasts on the polymers, flow cytometry-based vitality and proliferation assays were carried out.

Thirdly, as cell adhesion to the scaffold surface is a basic requirement for achieving a tight bone to scaffold contact [20], the adhesion strength of osteoblasts to the different polymers was tested using the atomic force microscopy based single cell force spectroscopy (SCFS) as recently reported [21].

The overall aim of this study was to find an implant coating material that fulfills several properties like high biocompatibility, strong cell adhesion, and the absence of coating-related pore occlusion at once. Against this background, this material may be further tested in future investigations to consider its suitability to act as a deposit and release system for drug delivery.

## 2. Materials and Methods

### 2.1. Osteoblast Isolation and Cultivation

As previously described, adult C57Bl6 mice or GFP*C57Bl6 mice were used for osteoblast isolation [19]. After mincing the calvarias of ten mice, these were dissolved in 5 mL Hank’s medium (HBSS, PAA Laboratories GmbH, Pasching, Austria) containing 200 U/mL collagenase II (Cell Systems, Troisdorf, Germany) and incubated five times for 10 min at 37 °C for proper digestion. Between every incubation step, the supernatant containing detached cells was collected and the remaining calvarial material was dissolved in new collagenase solution. Collected supernatants from all five incubation steps were pooled and centrifuged for 7 min at 1200 rpm before washing the resulting cell pellet twice with Dulbecco’s modified Eagle Medium (DMEM) (Biochrom, Berlin, Germany). For cultivation, the osteoblasts were seeded into T25 tissue culture flasks (TPP, Trasadingen, Switzerland) filled with 5 mL DMEM containing 10% foetal calf serum (FCS) (Biochrom, Berlin, Germany). The medium was changed twice a week and cells were split after reaching 80% confluency.

### 2.2. Selective Laser Melting of Titanium Scaffolds

**Figure 1:**
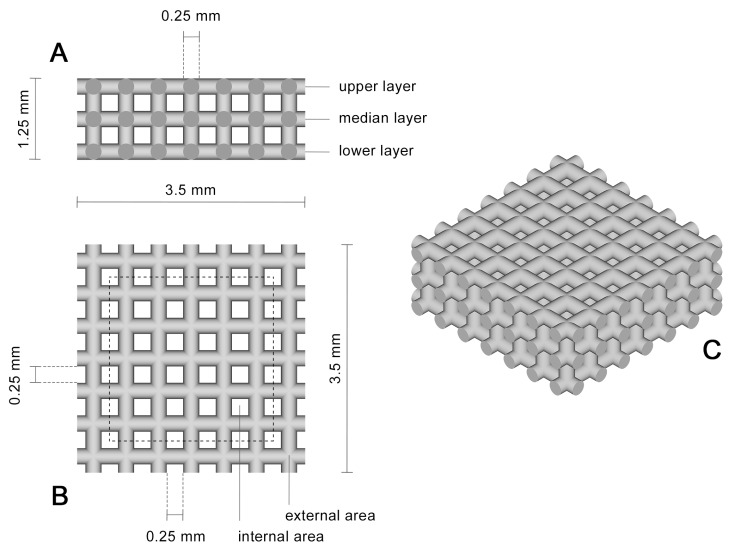
Schematic side view (**A**) top view (**B**) and 3D view of the scaffold geometry; (**C**) The dotted line marks the division of the implant into an external and internal area.

An SLM^®^ 280^HL^ Selective Laser Melting machine system (SLM Solutions GmbH, Lübeck, Germany) was used to create three-layered porous titanium scaffolds out of TiAl6V4 alloy with a length and width of 3.5 mm, height of 1.25 mm, and with a pore size as well as a strut width of 250 µm [5] (Figure 1). All scaffolds were post-treated using a chemical deburring process in order to create a smooth scaffold surface [18].

### 2.3. Viscosimetry of PCL and P(3HB) Coating Solutions

To determine the dynamic viscosity, an Ubbelohde viscosimeter (CT52 with Visco Clock, Schott Instruments GmbH, Mainz, Germany) was used with different capillary sizes, according to the viscosity of the solvent. Temperature was regulated at 20.0 ± 0.01 °C. Mass density of the solvent was calculated by defined volume in a graduated flask, and mass analyses were performed using a Kern 770 analytical precision balance (Gottlieb Kern & Sohn GmbH, Balingen-Frommern, Germany). The dynamic viscosity was determined via η = ν × ρ and ν = K × t with ν = kinematic viscosity, ρ = density of the coating solution, t = efflux time, and K = capillary constant. The measurement was repeated three times.

### 2.4. PCL and P(3HB) Coating of Titanium Scaffolds

In order to apply polymeric coatings to the porous titanium scaffolds, a manual dip-coating process was established using a constructed sample holder [17]. The scaffolds were immersed six times in 0.4 wt. % chloroformic solutions of either PCL or P(3HB), with an intermediate drying step being performed after each dipping process for 10 min at 23 ± 2 °C. After the final drying step, the scaffolds were dried in a vacuum cabinet drier at 40 °C and 40 mbar for seven days.

### 2.5. Characterisation of PCL and P(3HB) Coatings via ESEM and EDX Analysis

In order to gain an overview of the coated implant surface and to verify the coating, environmental scanning electron microscopy (ESEM) and energy-dispersive X-ray (EDX) analyses were performed using a scanning electron microscope (Quanta FEG 250, FEI, Eindhoven, The Netherlands) equipped with an EDX analysis unit [18]. After fixing PCL- and P(3HB)-coated scaffolds as well as uncoated ones (positive control), electron micrographic examinations were performed at 50 Pa and 10 kV in a moisturised atmosphere. For analysing of the pore diameter, the arithmetical means and the standard deviation were calculated from the maximum width of each pore (*n* = 35 per scaffold). Thereby occluded pores were also taken into account with a value of 0 µm. Additionally, EDX measurements were taken to determine the atomic composition of Ti and C elements on the scaffold’s surface.

### 2.6. Cross-Sections of PCL-Coated Titanium Scaffolds

**Figure 2:**
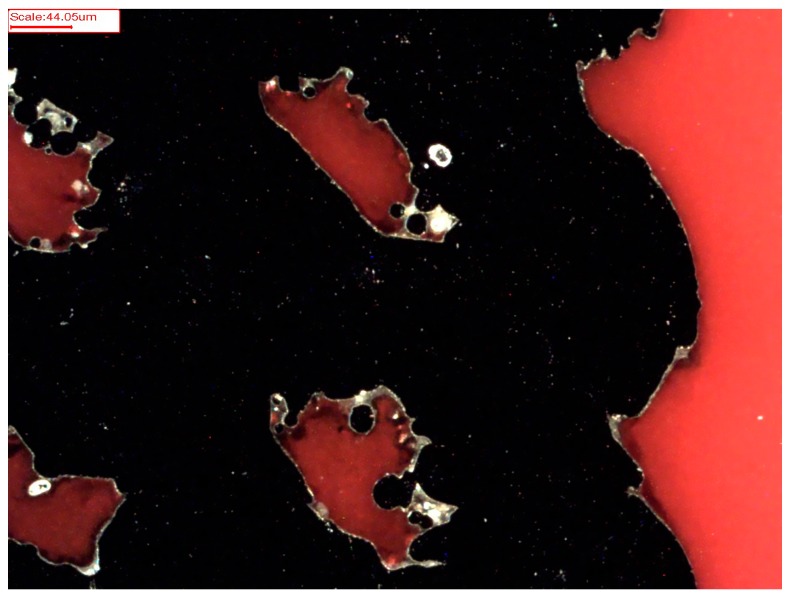
Exemplary micrograph image of a poly-ε-caprolactone (PCL)-coated titanium scaffold after cross-section preparation (black = titanium with pores; white rims = PCL coating; red = embedding medium; scale bar = 44.05 µm).

To gain a closer view of the coating efficiency throughout the whole implant, geometry cross-sections of PCL-coated titanium implants (*n* = 3) were prepared. The scaffolds were embedded in EppColor^TM^ Epoxy Resin with EpoColor Epoxy Hardener (ratio 5:1, Buehler, Lake Bluff, IL, USA) for 5 h at room temperature. Embedded scaffolds were grinded to the area of interest using a grinding machine (TegraPol-15, Struers GmbH, Willich, Germany) equipped with grinding SiC films (Struers GmbH, Willich, Germany). After grinding with a grain size of 1200, the samples were rotated about 90° and the grain size was changed to 2000. The second grinding step was performed until scratches resulting from the former grain size had completely disappeared. Afterwards, these steps were replicated using a grain size of 4000. Finally, the surface was polished using a Micro Floc disc with MasterPrep™ and MasterMet™2 polishing liquid (ratio 1:1, Buehler, Lake Bluff, IL, USA). Coating thickness was analysed using a calibrated microscope (Axioskop, Carl Zeiss AG, Oberkochen, Germany). Therefore, coating thickness was determined at different areas of the scaffold (see Figure 1). A representative image of a micrograph can be seen in Figure 2. For each area, at least three measurements were performed. Since P(3HB) turned out not to cover the metallic base body efficiently in ESEM and EDX analyses (see Section 3.2), cross-section preparation was dispensed with for this kind of coating.

### 2.7. Preparation and Characterisation of PCL and P(3HB) Foils

Following the procedure according to Wulf et al. [22], 1 g PCL (Capa™ 6800, Perstorp, Warrington, UK) or 1 g P(3HB) (Helmholtz-Zentrum für Umweltforschung, UFZ, Leipzig, Germany) were dissolved in 25 mL chloroform to fabricate polymer sheets. After several washing steps (two days in methanol, two days in distilled water, three times for 60 min each in 0.05% Tween 20, three times for 60 min each in distilled water), the sheets were dried in a vacuum cabinet drier at 40 °C and 40 mbar for seven days. In order to be able to use them in a circular form in vitality and proliferation assays, foils with a diameter of 6.4 mm were stamped out of the polymer sheets, sterilised using 70% ethanol, and air-dried under laminar flow. In order to characterise the foils’ surface, ESEM analyses were performed. 

### 2.8. Vitality and Proliferation Assays

To evaluate the osteoblast’s vitality and proliferation behaviour on the different materials, the CFSE Proliferation Kit (Life Technologies, Darmstadt, Germany) was used, following the manufacturer’s guidelines.

#### 2.8.1. CFSE Staining of Osteoblasts 

The 1 × 10^6^ murine osteoblasts (P9 to P10) were trypsinised and resuspended in 500 µL prewarmed phosphate buffered saline (PBS) with 1% bovine serum albumin (BSA) (Biochrom AG, Berlin, Germany). After adding 5 µM carbofluorescein succinimidyl ester (CFSE), the solution was mixed gently and incubated for 10 min at 37 °C to achieve a fast and even staining. In order to stop the staining process, 5 mL of cold (stored on ice beforehand) DMEM with 10% FKS was added and kept in ice water for 5 min. To remove surplus staining reagent, the suspension was centrifuged (10 min at 1000 rpm) and washed three times with culture medium. 

#### 2.8.2. Vitality and Proliferation Assay on Polymer Foils

**Figure 3:**
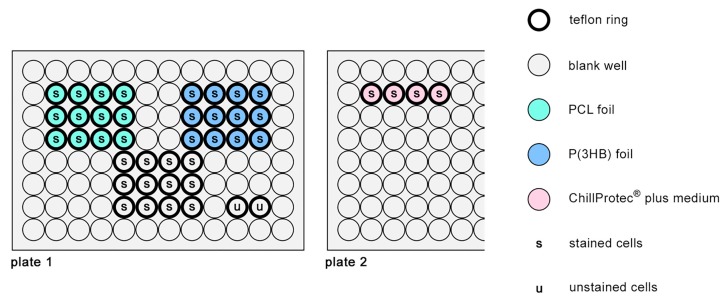
Experimental set-up for vitality and proliferation assays on PCL and poly(3-hydroxybutyrate) (P(3HB)) foils.

For testing the polymer’s biocompatibility (experimental set-up shown in Figure 3), each of the 12 sterilised (70% Ethanol for 5 min) PCL and P(3HB) foils (6.4 mm in diameter, thickness 154 µm ± 12 µm (PCL) and 115 µm ± 24 µm (P(3HB)) were inserted in the bottom of the wells of a cell culture-treated flat-bottomed 96 well plate (Figure 3; plate 1). Due to technical reasons, during the entire experiment (stamping, storage, sterilisation, and handling), the foils randomly faced the well bottom with their original upper or lower side. In order to avoid flotation of the foils within the cell culture medium, these were kept down by custom-designed Teflon rings also hindering cells from migrating to the well bottom underneath. As a positive control for cell growth, 12 blank wells were also equipped with Teflon rings to ensure a similar growing area. After adding 150 µL prewarmed DMEM with 10% FCS to each prepared well, 1 × 10^4^ CFSE-stained osteoblasts (Figure 3; s) were gently placed at the bottom of these wells. In order to test for a possible autofluorescence of the osteoblasts in the FL-1 channel (used for CFSE detection) within the later proliferation analysis of the samples, two blank wells were equipped with Teflon rings and 2 × 10^4^ unstained cells (Figure 3; u) in 150 µL prewarmed DMEM with 10% FCS. To detect a starting point of proliferation (generation 0), 1 × 10^4^ stained cells were inserted into four blank wells of another well plate (Figure 3; plate 2) also equipped with Teflon rings, each being filled with 150 µL ChillProtec plus medium^®^ (Merck Millipore, Darmstadt, Germany). The latter keeps cells alive at low temperatures while preventing them from proliferating by decreasing their metabolic activity. Plate 1 (PCL, P(3HB), positive control, and unstained cell control) was incubated for three days at 37 °C and 5% CO_2_ while plate 2 (generation 0) was stored at 4 °C for the same time period. This experimental set-up was performed three times.

#### 2.8.3. Vitality and Proliferation Assay on Titanium Scaffolds

**Figure 4:**
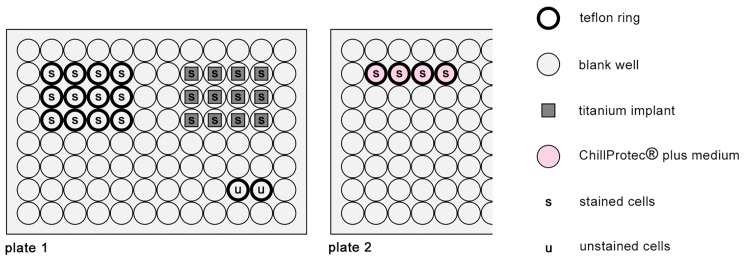
Experimental set-up for vitality and proliferation assays on titanium scaffolds.

To test the biocompatibility of the titanium scaffolds (experimental set-up shown in Figure 4), 12 sterilised titanium scaffolds were each inserted at the well bottoms of a cell culture-treated flat-bottomed 96 well plate (Figure 4; plate 1). As a positive control for cell growth, 12 blank wells were equipped with Teflon rings (see Section 2.8.2) to ensure the same growing conditions for the positive controls of both experimental set-ups. As previously described (see Section 2.8.2), the prepared wells were filled with cell culture medium and populated with stained osteoblasts (Figure 4; s). While 1 × 10^4^ cells were used for the positive control, 2.5 × 10^4^ osteoblasts were seeded on each titanium scaffold to ensure a similar cell number growing on both the scaffold and the positive control. Before performing the assays, pre-tests were run to determine an acceptable seeding concentration with 2.5 × 10^4^ cells per implant being defined as most suitable for comparing the scaffolds with the flat well surface area. An unstained cell control (Figure 4; u) was added to the same plate (see Section 2.8.2), as well as a generation 0 control on a second plate (Figure 4; plate 2). Plate 1 (titanium scaffolds, positive control, and unstained cell control) was incubated for three days at 37 °C and 5% CO_2_ while plate 2 (generation 0) was stored at 4 °C for the same time period. This experimental set-up was performed twice.

#### 2.8.4. Data Analysis

After incubation, the medium was removed from the wells and any remaining FCS was washed away using PBS. The cells were then trypsinised and within one material type (PCL, P(3HB), titanium, and positive control) the cell suspensions from four adjacent wells were pooled in one sample, resulting in three samples each per material type per experiment. The four wells with cells in generation 0 as well as the two wells with unstained cells were each pooled to one control sample. Before analysing the samples with an FACScalibur flow cytometer and the flow cytometry analysis software BD CellQuest^TM^ Pro (BD, Heidelberg, Germany), the collected cells were stained using TO-PRO^®^-3 (Life Technologies, Darmstadt, Germany), which invades the cytoplasm through the porous cell membrane of damaged or dead cells to finally stain the nucleus. For further processing, the raw data were analysed using FlowJo flow and image cytometry analysis software. Before determining the living fraction of actual cells by using the TO-PRO^®^-3 concentration histogram (visible in FL-4 channel), cell detritus was gated out in an FSC/SSC dot plot. For proliferation analysis, the starting point of proliferation was set at the peak of the highest CFSE concentration in the histogram (visible in FL-1 channel) of the generation 0 control, and the proliferation index of each sample was determined using the proliferation function.

### 2.9. Live Cell Imaging 

In order to analyse the cell growth behaviour of osteoblasts on the different materials over a time period, Live Cell Imaging (LCI) was performed. Each three titanium scaffolds coated with either PCL or P(3HB) as well as three uncoated titanium scaffolds (positive control) were inserted into the wells of a flat-bottomed 96 well plate. After adding 150 µL prewarmed DMEM with 10% FCS to each prepared well, a cell concentration of 2.5 × 10^4^ GFP (Green Fluorescent Protein)-linked osteoblasts (P 9) was settled gently on top of each scaffold and incubated at 37 °C and 5% CO_2_ for proper adhesion. For visualisation using an inverse microscope DMI6000 B (Leica Microsystems GmbH, Wetzlar, Germany), the scaffolds had to be turned upside down. Therefore, the scaffolds were moved to other wells equipped with prewarmed 150 µL DMEM with 10% FCS and a custom-designed circular Teflon construction as previously described [19]. The latter ensured a certain distance between the populated scaffold surface and the well bottom in order to avoid a migration of cells. Using LAS AF 2.6.0 microscope imaging software (version 2.6.0, Leica Microsystems GmbH, Wetzlar, Germany), two pictures were taken from a representative spot of each scaffold every 15 min: To visualise the scaffold surface, a transmitted light camera was used, while a fluorescence camera captured the green fluorescent GFP-linked osteoblasts. The cells were observed for seven days under cell culture conditions at 37 °C and 5% CO_2_, the culture medium being substituted once on day three. Cell count and populated area were analysed by Wimasis Image Analysis GmbH (Munich, Germany). As the outcoming data were defined as cells or populated area per picture, the pore area was subtracted from the whole picture using ImageJ picture analysis software (Fiji vers. 2.0.0, National Institutes of Health (NIH), Bethesda, MD, USA), resulting in more comparable results between the different visualised spots on the scaffolds.

### 2.10. Single Cell Force Spectroscopy

To analyse the attachment forces between osteoblasts and the polymer surfaces, Single Cell Force Spectroscopy (SCFS) was performed using an atomic force microscope (AFM; NanoWizard II, JPK–Instruments AG, Berlin, Germany). A cantilever (Arrow–TL1, Nanoworld AG, Neuchatel, Switzerland; nominal force constant of 0.03 N/m) was coated with human plasma-derived fibronectin (Biochrom AG, Berlin, Germany; diluted in PBS to an end concentration of 0.15 mg/mL) for 30 min at 37 °C in a humid environment in order to enable a strong adhesion of single osteoblasts. Beforehand, the surfaces of petri dishes (Biochrom/TPP, Berlin, Germany; 34 mm in diameter) were partially spray coated with PCL and P(3HB), creating a cell attachment platform with three different surface types (PCL, P(3HB), and uncoated). As the dishes were cell culture-treated, the uncoated surface functioned as a positive control. For proper performance of the single cell force microscopy, the inspected ground surface had to be see-through in order to track, catch, and examine single cells with the cantilever while being constantly observed through a microscope. Whereas opaque titanium scaffolds were not suitable for this kind of investigation, the transparent yet flat petri dishes were chosen to act as a ground surface for these experiments. After sterilising (70% ethanol for 5 min), a dish filled with 2 mL of medium (96% CO_2_-independent medium, 2% stable glutamine (Biochrom AG, Berlin, Germany), 2% FCS) was warmed up to 37 °C by a petri dish heater (PDH, JPK–Instruments AG) before gently inserting the coated cantilever. In order to avoid large thermal drifts during the experiment, the cantilever was allowed to rest for at least 45 min. Using a standard protocol, murine osteoblasts (P 11) were trypsinised and a small amount (ca. 100 µL) of cell suspension was added to the petri dish filled with medium. Before measuring, a single non-adherent round osteoblast was tracked using an inverted optical microscope (Axio observer D1, Zeiss, Jena, Germany) and caught with the cantilever tip by pressing it onto the cell for 10 s with a constant force of 2 nN. To ensure that the cell was properly attached to the fibronectin, the cantilever remained floating for 10 min before starting the experiments. The single osteoblast was allowed to attach for 180 s under a constant force of 1 nN before retracting it from the petri dish bottom while measuring the forces between the cell and the dish surface. After detaching the cell from the bottom of the dish, the measurement was stopped and repeated with the same cell (still attached to the cantilever tip) on PCL- and P(3HB)-coated areas of the petri dish. In addition to these three measurements, the same cell was measured again on all three surface types using an attachment time of 5 s. Due to a loss of cells during the single measurements within one experiment, not all of a total of 14 cells could be measured on every surface type after every attachment time (Table 1).

**Figure 5:**
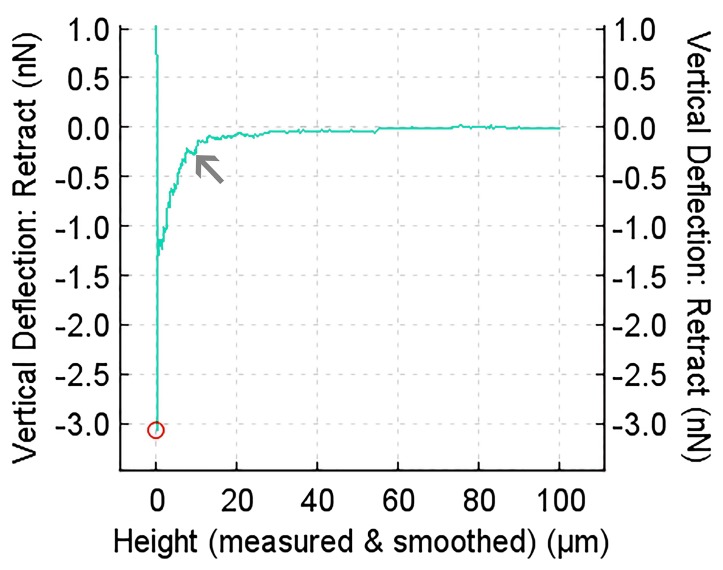
Force-displacement (FD) curve of a single osteoblast attached to PCL-coated dish surface for 180 s. The curve shows the maximum detachment force (red circle) of the cell and single cell-PCL surface bindings that were separated by moving the cell away from the PCL surface (small force steps, arrow).

JPK SPM software (JPK–Instruments AG, Berlin, Germany; vers. 4.3.25) was used for analysing the force-displacement curves. Cell adhesion was determined by the maximum peak of the retraction curve (Figure 5; red circle).

### 2.11. Statistical Analysis

Statistical analyses of data were performed using SAS^®^ software, Version 9.3 (SAS Institute Inc., Cary, NC, USA). Type I error was set at 5%, so *p*–values < 0.05 were considered statistically significant. The statistical analysis for LCI was performed using the Ryan-Einot-Gabriel-Welsh Multiple Range Test and the two-sample *t*-test. Additionally, a linear regression analysis was performed, followed by the comparison of the regression coefficients using the *F*-test for interaction between time and material. Since the data from the vitality and proliferation assays were not normally distributed, the Kruskal–Wallis Test was performed followed by the Wilcoxon’s signed-rank test. The same tests were used to compare the not normally distributed Single Cell Force Spectroscopy results.

## 3. Results

### 3.1. Viscosimetry of PCL and P(3HB) Coating Solutions

Viscosity analysis resulted in a mean value for dynamic viscosity of 1.53 Nsm^−2^ for PCL and 2.26 Nsm^−2^ for P(3HB).

### 3.2. ESEM and EDX Analysis of PCL and P(3HB) Coatings

**Figure 6:**
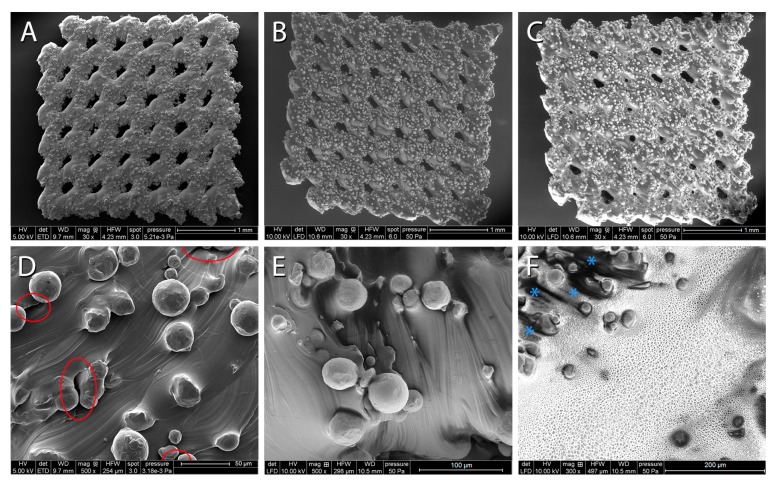
Representative environmental scanning electron microscopy (ESEM) micrographs of uncoated (**A**,**D**), PCL-coated (**B**,**E**) and P(3HB)-coated (**C**,**F**) porous titanium scaffolds in overview (**A**–**C**; scale bar = 1 mm) and in detail (**D**–**F**; scale bar = 50 µm (uncoated), 100 µm (PCL), 200 µm (P(3HB)). Within the red circles cavities between microparticles are shown on the uncoated titanium scaffolds, whereas the blue asterisks mark uncoated titanium after applying P(3HB) coating.

Titanium scaffolds coated with either PCL or P(3HB) as well as uncoated scaffolds were visualised via ESEM (Figure 6). In overview pictures (Figure 6A–C) on PCL-coated scaffolds, all pores were still open while being smaller due to polymeric coverage of the struts. In contrast, P(3HB)-coated scaffolds showed several sealed pores. The measurement of the pore diameters revealed similar values for uncoated and PCL-coated titanium scaffolds, whereas P(3HB)-coated ones showed a clearly lower pore diameter with an exceptionally high standard deviation caused by 11 of the 35 measured pores being occluded after coating (Table 2). In the close-up view (Figure 6D–F), uncoated titanium scaffolds showed a surface with many SLM-induced micro-particles. Partially fused micro-particles resulted in several cavities (Figure 6D; red circles), which featured a particular obstacle for the polymer coating. After applying PCL coating, the surface with prominent micro-particles was completely covered, filling out even the cavities between the microparticles, whereas the P(3HB) coating resulted in a brittle, porous, and inhomogeneous surface leaving several microparticles, cavities, and even larger titanium surface areas uncoated (Figure 6F; blue asterisks). 

Additionally performed EDX measurements (Table 3) revealed a considerable increase in the atomic percentage of carbon for both coatings in comparison to the uncoated control. Regarding the atomic percentage of titanium, the PCL-coated scaffold showed a lower value than P(3HB), which indicated a more complete coating success using PCL.

### 3.3. Cross-Sections of PCL-Coated Titanium Scaffolds

Nearly the same PCL thickness of 4.1 µm to 4.8 µm was observed over the entire implant geometry (Table 4). Only the upper layer of the internal area revealed a lower average thickness of 1.5 µm. Overall, a huge standard deviation of approximately 50% of the arithmetic mean was calculated, possibly caused by the structurally varying surface of the scaffolds. Furthermore, the observations revealed a gapless coating with tight titanium-PCL interface.

### 3.4. ESEM Analysis of PCL and P(3HB) Foils

**Figure 7:**
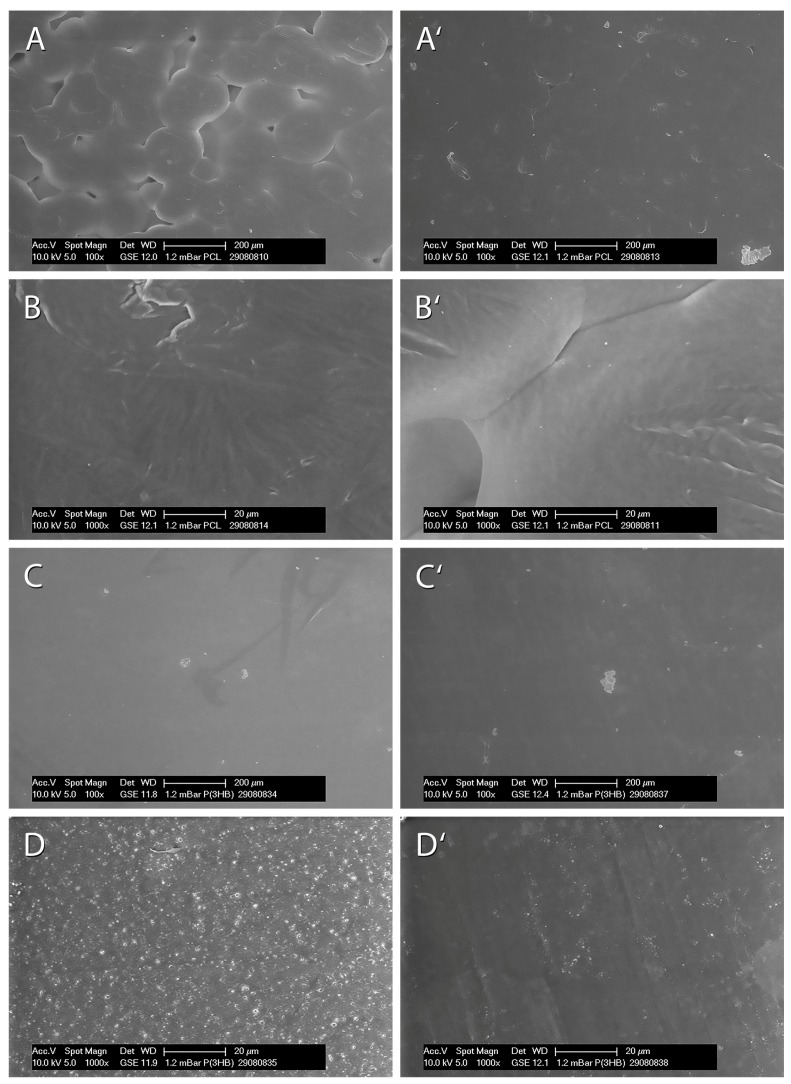
Representative ESEM micrographs of PCL ((**A**,**B**) first side and (**A**’,**B**’) second side) and P(3HB) ((**C**,**C**’) fist side and (**D**,**D**’) second side) foils in overview ((**A**,**A**’) and (**C**,**C**’); 100-fold magnification, scale bar: 200 µm) and in detail ((**B**,**B**’) and (**D**,**D**’); 1000-fold magnification, scale bar: 20 µm).

Prepared polymer foils for the Vitality and Proliferation Assays were analysed using ESEM measurements (Figure 7). In overview pictures of 100-fold magnification, PCL foils (Figure 7A,A’) showed typical spherulitic structures, which rose towards their centre. Between these structures, irregular cavities were observed. In contrast, the surface of P(3HB) foils appeared smooth and homogeneous in the overview pictures (Figure 7C,C’). In the close-up view of 1000-fold magnification, PCL foils (Figure 7B,B’) showed an irregular streaky microstructure within the spherulitic shapes, while P(3HB) foils (Figure 7D,D’) revealed a regular grainy microstructure on one side (cavities of about 2 µm; Figure 7D) and an irregular one on the other side (cavities of about 0.8 µm; Figure 7D’).

### 3.5. Vitality and Proliferation Assays

**Figure 8:**
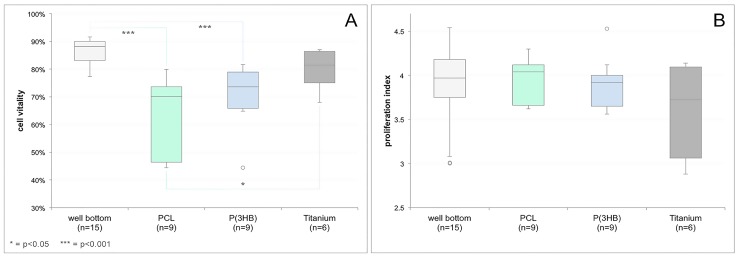
Vitality of green fluorescent protein (GFP)-osteoblasts on the blank well bottom (positive control), PCL and P(3HB) foils, as well as on titanium scaffolds (**A**). The Kruskall–Wallis Test followed by the Wilcoxon’s rank-sum test showed a significantly lower cell vitality on both PCL and P(3HB) compared to the well bottom. Also, significantly lower cell vitality on PCL compared to the titanium scaffolds was revealed. Proliferation index of GFP-osteoblasts on the blank well bottom (positive control), PCL and P(3HB) foils, as well as on titanium scaffolds (**B**). No significant difference between the materials could be found using the Kruskall–Wallis Test (horizontal line = median, whiskers = minimal and maximal value, circle = outlier, * = *p* < 0.05, *** = *p* < 0.001).

Regarding the osteoblast vitality on the different materials, median values of 88.19% (std = standard deviation = 4.68%) for well bottom, 81.47% (std = 7.79%) for titanium, 70.12% (std = 14.83%) for PCL, and 73.63% (std = 11.53%) for P(3HB), respectively, were detected (Figure 8A). The assays showed a significantly lower vitality of the osteoblasts growing on PCL in comparison to the well bottom (*p* = 0.0002) and the titanium scaffolds (*p* = 0.0392), while the vitality on P(3HB) was only significantly lower in comparison to the well bottom (*p* = 0.0004). The proliferation index (as defined as the mean number of cell divisions of all proliferating cells) showed median values of 3.97 (std = 0.48) for the well bottom, 3.73 (std = 0.58) for titanium, 4.04 (std = 0.27) for PCL, and 3.92 (std = 0.30) for P(3HB), respectively (Figure 8B). No significant difference between the different materials could be detected. The values for vitality and proliferation index of the single samples as well as the median values for each material and the associated standard deviations can be found in the supplementary materials section (Table S1).

### 3.6. Live Cell Imaging

**Figure 9:**
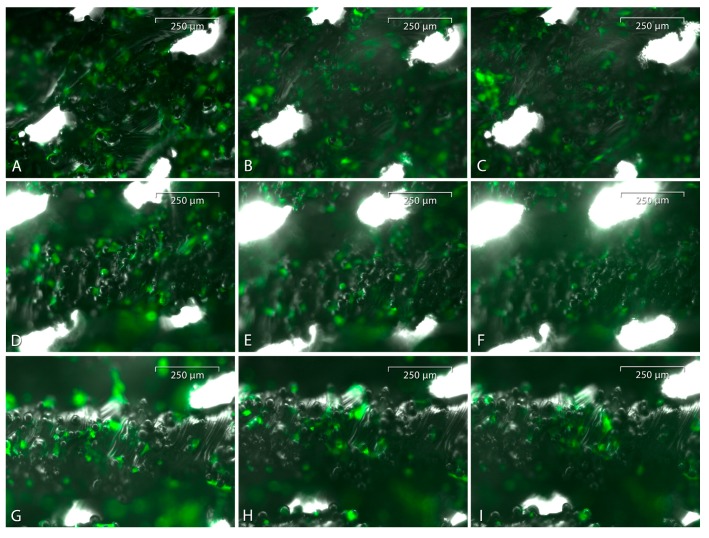
GFP-linked osteoblasts on an uncoated (**A**–**C**), PCL-coated (**D**–**F**) or P(3HB)-coated (**G**–**I**) titanium scaffold on day 0 (**A**,**D**,**G**), 3 (**B**,**E**,**H**) and 7 (**C**,**F**,**I**). The pictures were taken at a 10-fold magnification (scale bar: 250 µm).

Judging from the taken pictures, semiquantitatively the cell population density on the uncoated titanium scaffolds seemed to remain stable from day 1 to 7, while maintaining a flat and attached cell morphology (Figure 9A–C). However, on the coated scaffolds, the osteoblasts seemed to detach themselves and disappear over time due to the loss of GFP signal (Figure 9D–I).

**Figure 10:**
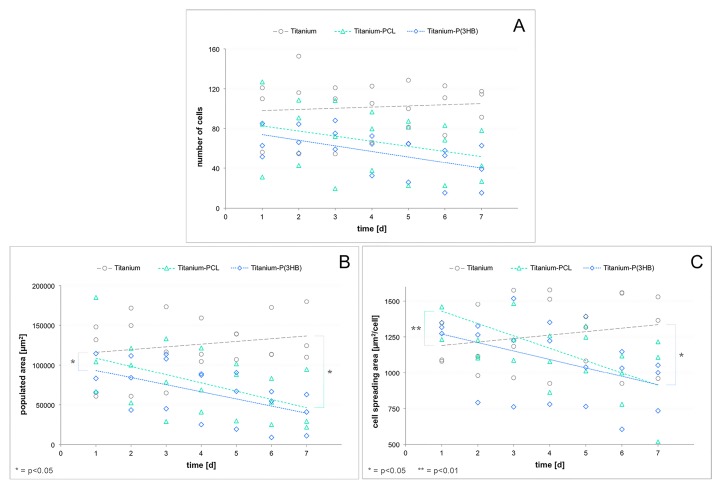
Number of GFP-osteoblasts on uncoated, PCL-coated and P(3HB)-coated titanium scaffolds in the course of 7 days (**A**), as well as the populated area of these implants (**B**) and the cell spreading area of the osteoblasts (**C**) in the same temporal dimensions. Using the *F*-test for interaction between time and materials, significant differences could be revealed between the populated area of uncoated and both PCL- and P(3HB)-coated scaffolds. Also, significant differences could be found for the cell spreading area of uncoated scaffolds compared to coated ones (circles = single values for uncoated titanium scaffolds, triangles = single values for PCL-coated scaffolds, rhombi = single values for P(3HB)-coated scaffolds, lines = regression curves, * = *p* < 0.05, ** = *p* < 0.01).

The calculated data was presented as number of cells or populated area on 1 mm^2^ of implant surface. Additionally, the cell spreading area (populated area divided by the number of cells = average size of an osteoblast) as a parameter of cell attachment was calculated. The median values as well as the associated standard deviations regarding each of the calculated parameters for each material on each day can be found in the supporting material (Table S2). On day 7, the Ryan-Einot-Gabriel-Welsh Multiple Range Test and the two-sample *t*-test showed a significantly higher number of cells growing on the uncoated titanium scaffolds in comparison to both PCL- and P(3HB)-coated ones (graph A in Figure S1). It also revealed a significantly higher populated implant surface on the uncoated scaffolds in comparison to both polymer coatings on days 6 and 7 (graphs B and C in Figure S1). Regression analyses showed a rather stable to increasing number of cells over time on uncoated scaffolds, while the cell numbers on both coating materials decreased during the days of observation (Figure 10A). Similar results could be seen for the populated scaffold area and the cell spreading area, thus indicating a detachment and dying of osteoblasts on PCL- and P(3HB)-coated titanium scaffolds over time (Figure 10B,C). In order to statistically compare the calculated regression coefficients, the *F*-test for interaction between time and materials was performed. Looking at the populated area and the cell spreading area, significant differences between the uncoated and both PCL- and P(3HB)-coated scaffolds could be found, while no significant changes were observed for the number of cells.

### 3.7. Single Cell Force Spectroscopy

**Figure 11:**
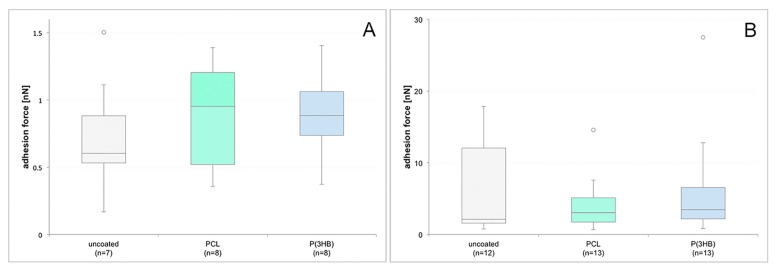
Number of GFP-osteoblasts on uncoated, PCL-coated and P(3HB)-coated titanium scaffolds in the course of 7 days (**A**), as well as the populated area of these implants (**B**) and the cell spreading area of the osteoblasts (**C**) in the same temporal dimensions. Using the *F*-test for interaction between time and materials significant differences could be revealed between the populated area of uncoated and both PCL- and P(3HB)-coated scaffolds. Also, significant differences could be found for the cell spreading area of uncoated scaffolds compared to coated ones (circles = single values for uncoated titanium scaffolds, triangles = single values for PCL coated scaffolds, rhombi = single values for P(3HB)-coated scaffolds, lines = regression curves).

SCFS was performed in order to evaluate the adhesion of single osteoblasts to the different materials quantitatively. By averaging the maximum detachment forces of each cell, the adhesion force for a specific material was determined. Since the polymer surfaces were slightly uneven, the results showed high variance in cell adherence strength, x–axis scale, and magnitude. After an attachment time of 5 s (Figure 11A), median values of 0.6 nN (std = 0.44 nN) for the uncoated petri dish, 0.95 nN (std = 0.41 nN) for PCL, and 0.88 nN (std = 0.33 nN) for P(3HB) were detected, while the median values after an attachment time of 180 s (Figure 11B) were 2.12 nN (std = 6.34 nN) for the uncoated petri dish, 3.04 nN (std = 3.76 nN) for PCL, and 3.45 nN (std = 7.21 nN) for P(3HB), respectively. Using the Wilcoxon’s signed-rank test, the SCFS investigations revealed no significant changes in osteoblast adhesion to the polymer surfaces compared with each other and with the uncoated petri dish surface after each attachment time. The values for adhesion strength of the single osteoblasts as well as the median values for each material at each adhesion time and the associated standard deviations can be found in the supplementary materials section (Table S3).

## 4. Discussion 

Synthetic PCL and biologically generated P(3HB) were tested for coating efficiency, biocompatibility, and cell adhesion in order to prove their suitability as coating materials for porous titanium scaffolds to possibly act as a deposit and release system for growth factors. In terms of coating efficiency, PCL showed a smooth and complete coating of the underlying titanium in ESEM and EDX analyses. In contrast, P(3HB) coating was incomplete, resulting in bare titanium areas both visible in ESEM pictures and EDX data. Since a low viscosity leads to a deeper pore penetration [23], PCL coating could be performed without any pore occlusion, resulting in a complete coating even inside the scaffold with little variation of coating thickness and nearly the same pore diameter as uncoated titanium implants. On the other hand, P(3HB) coating resulted in several occluded pores and thereby in a decrease in pore diameter and overall implant porosity. According to recent studies, factors like osteoblast differentiation and proliferation, and thereby osteointegration, increase with higher implant porosity [24,25]. Thus, it appears that PCL is more suitable as an implant coating material for bone implants than P(3HB). In contrast, both polymers showed similar results regarding their biocompatibility using LCI with GFP-osteoblasts seeded on PCL- and P(3HB)-coated titanium scaffolds. Nevertheless, both polymer coatings resulted in a decrease in cell number and populated areas on the implants as well in a progressive detachment of cells from the coating surface (cell spreading area), wheareas all parameters increased on titanium. Since the polymer solutions used for scaffold coating contained chloroform, which is reported to have cytotoxic effects [26,27], this phenomenon might be caused by remaining residues from the coating process. Due to the fact that the culture medium was only substituted once on day three within the seven days of observation, these residues possibly dissolved within the culture medium and accumulated over the three days, sustainably affecting the osteoblast’s vitality and behaviour. Similar differences were reported between uncoated and PCL-coated titanium scaffolds within an angiogenesis study in which the scaffolds were also incubated for several days without medium substitution [11]. Also, since the hydrophobic polymer surfaces were reported to result in low cell adhesion [28], it might be harder for cells to attach to and be maintained on the inverted coated scaffolds than on the bare ones. In comparison, this would explain the rather high values within performed vitality and proliferation assays since the polymer foils were not inverted. Looking at the osteoblast proliferation indices, no significant differences could be detected. Due to the fact that osteoblast proliferation includes interaction between cellular integrins and extracellular matrix proteins (ECMPs) such as fibronectin, osteopontin, and collagen type I [29], the binding to the polymer surfaces might be performed indirectly [30]. In contrast, regarding the osteoblast’s vitality, the assays showed significantly lower values for both PCL and P(3HB) compared to the well bottom. However, these differences should not be weighted too strongly since the well bottom was specially treated in order to serve as a surface for cell growth. Thereby, it is not surprising that even decent vitality values on polymer surfaces (median on PCL = 70%, median on P(3HB) = 75%) showed significant differences to the positive control. Thus, the greater focus lies on the comparison of the polymers to the bare titanium scaffolds, which resulted in only significantly lower values for PCL. Nevertheless, since, in accordance with DIN EN ISO 10993-5:2009-10, vitality values of at least 70% result in a material being labeled as non-cytotoxic, both polymers fulfill these criteria regarding biocompatibility [31]. 

## 5. Conclusions

As a negative effect of chloroformic residues from the coating process on cell vitality and behaviour can not be ruled out, the overall experimental set-up should be extended. Namely, after the coating process, implants should be checked on chloroformic residues, resulting in additional purifying steps of the scaffolds in a positive case. In general, the usage of chloroform as a solvent for PCL does not pose a risk in terms of biocompatibility if the resulting polymeric structures are properly post-treated. Therefore, the content of chloroformic residues can be decreased under the mandatory value of 0.006 m% [32], neither affecting the polymer’s morphology nor its degree of crystallinity. Overall, within the given experimental set-up, both polymers showed acceptable values regarding their biocompatibility. However, since the use of PCL did not result in any pore occlusion, it is preferably suited as an implant coating material, having the potential to act as a deposit and release system for growth factors in future experiments.

## Figures and Tables

**Table 1:** Measured osteoblasts attached to different surface types after different attachment times.

Surface Type	Attachment Time	
	180 s	5 s
uncoated	12	7
PCL-coated	13	8
P(3HB)-coated	13	8

**Table 2:** Arithmetical means and standard deviations of the pore diameter analyses (*n* = 35).

Uncoated Porous Titanium Scaffolds	PCL Coated Porous Titanium Scaffolds	P(3HB) Coated Porous Titanium Scaffolds
230.8 ± 42.7 µm	217.7 ± 46.4 µm	88.7 ± 84.6 µm

**Table 3:** Energy-dispersive X-ray (EDX) data on surface composition (atomic percentage (At-%) for the relevant elements titanium (Ti) and carbon (C)) of the coated porous titanium scaffolds.

Scaffold Modification	At-% Ti	At-% C
Uncoated	93.74	6.26
PCL coated	3.90	96.10
P(3HB) coated	21.34	78.66

**Table 4:** Polymer layer thicknesses and standard deviations of cross-sections of PCL-coated titanium scaffolds (*n* = 3 and each area with 6 measurements).

Position	External Area	Internal Area
Upper layer	4.6 ± 2.0 µm	1.5 ± 0.9 µm
Median layer	4.1 ± 1.8 µm	4.8 ± 2.8 µm
Lower layer	4.4 ± 1.6 µm	4.5 ± 3.0 µm

## Supplementary Materials

The following are available online at www.mdpi.com/1996-1944/10/12/1344/s1. Table S1: Single values and standard deviations of vitality and proliferation assays. Table S2: Single values and standard deviations of live cell imaging experiments. Figure S1: Number of GFP-osteoblasts on uncoated, PCL-coated and P(3HB)-coated titanium scaffolds on day 7 (A) and populated area of these implants on days 6 (B) and 7 (C). Table S3: Single values and standard deviations of single cell force spectroscopy.
