# Supplementary figures and images for: In Vitro Evaluation of PCL and P(3HB) as Coating Materials for Selective Laser Melted Porous Titanium Implants
Source: Materials (Basel). 2017 Nov 23;10(12):1344. doi: 10.3390/ma10121344 (PMC5744279; doi:10.3390/ma10121344)

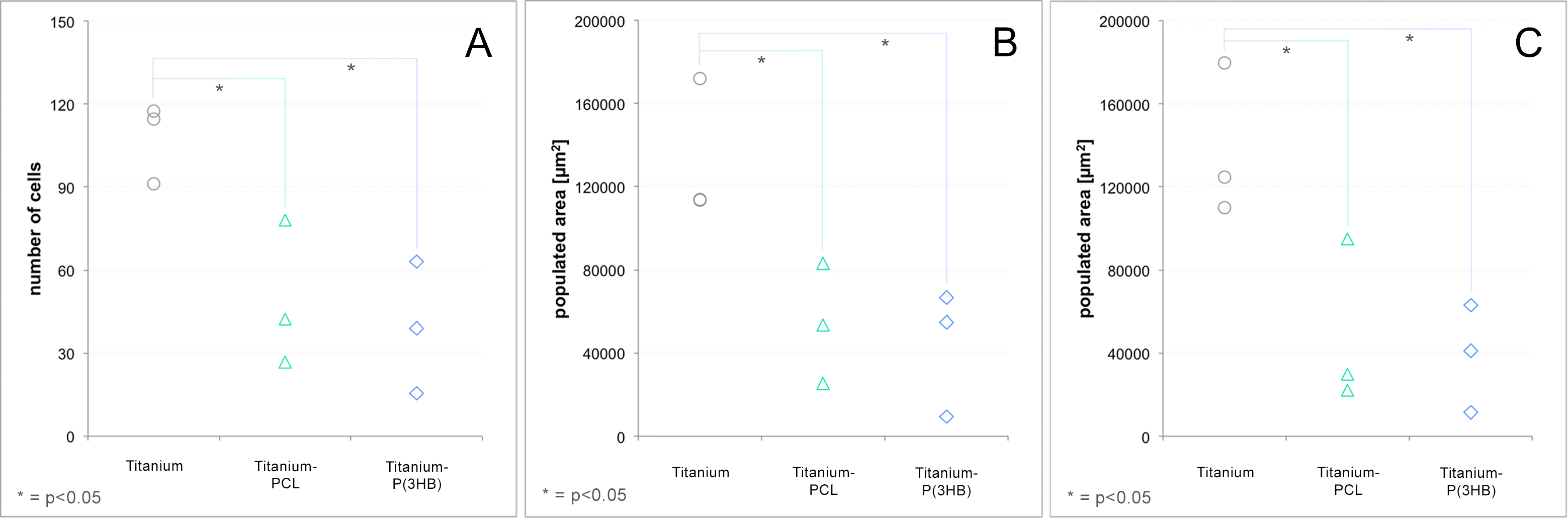

Supplement: Supplementary file 1 [file materials-10-01344-s001.zip › Figure S1.tif]
